# Breast tumor cell hybrids form spontaneously *in vivo* and contribute to breast tumor metastases

**DOI:** 10.1063/1.5024744

**Published:** 2018-08-07

**Authors:** Casey A. Chitwood, Claire Dietzsch, Gabriel Jacobs, Tanner McArdle, Brian T. Freeman, Annanya Banga, Felicite K. Noubissi, Brenda M. Ogle

**Affiliations:** 1 Department of Biomedical Engineering, University of Minnesota–Twin Cities, Minneapolis, Minnesota 55455, USA; 2 Stem Cell Institute, University of Minnesota–Twin Cities, Minneapolis, Minnesota 55455, USA; 3 Department of Biology/RCMI, Jackson State University, Jackson, Mississippi 39217, USA; 4 Department of Biomedical Engineering, University of Wisconsin–Madison, Madison, Wisconsin 53706, USA; 5 Masonic Cancer Center, University of Minnesota–Twin Cities, Minneapolis, Minnesota 55455, USA; 6 Lillehei Heart Institute, University of Minnesota–Twin Cities, Minneapolis, Minnesota 55455, USA; 7 Institute for Engineering in Medicine, University of Minnesota–Twin Cities, Minneapolis, Minnesota 55455, USA

## Abstract

Cancer cell fusion was suggested as a mechanism of metastasis about a century ago. Since then, many additional modes of material transfer (i.e., tunneling nanotubes, and exosomes) to generate cell hybrids have been identified. However, studies documenting spontaneous tumor hybrid formation *in vivo* as a mechanism that enables metastasis are still lacking. Here, we tested whether spontaneous hybrid formation *in vivo* contributes to bona fide metastatic tumors. We first used single cell RNASeq to analyze the gene expression profile of spontaneously formed cancer cell-stromal hybrids, and results revealed that hybrids exhibit a clustering pattern that is distinct from either parental cell and suggestive of substantial diversity of individual hybrids. Despite the newly gained diversity, hybrids can retain expression of critical genes of each parental cell. To assess the biological impact of cancer cell hybrids *in vivo*, we transfected murine mammary tumor cells, isolated from FVB/N-Tg(MMTV-PyVT)634Mul/J mice (PyVT) with *Cre* recombinase prior to injection to the murine fat pad of FVB.129S6(B6)-*Gt(ROSA)26Sor^tm^*^1*(Luc)Kael*^/J mice such that luciferase expression is induced with hybrid formation; luciferase expression was tracked for up to four months. We observed that hybrid formation occurs spontaneously *in vivo* and that a significantly higher number of hybrids reside in metastases compared to the primary tumor, supporting the possibility that hybrids can emerge from the primary tumor and proliferate to help create a new tumor at a distant site. Additional studies are now warranted to delineate the mechanisms of cancer cell hybrid transit to metastases since drugs to inhibit hybrid formation might prevent metastatic spread.

## INTRODUCTION

**FIG. 1.**
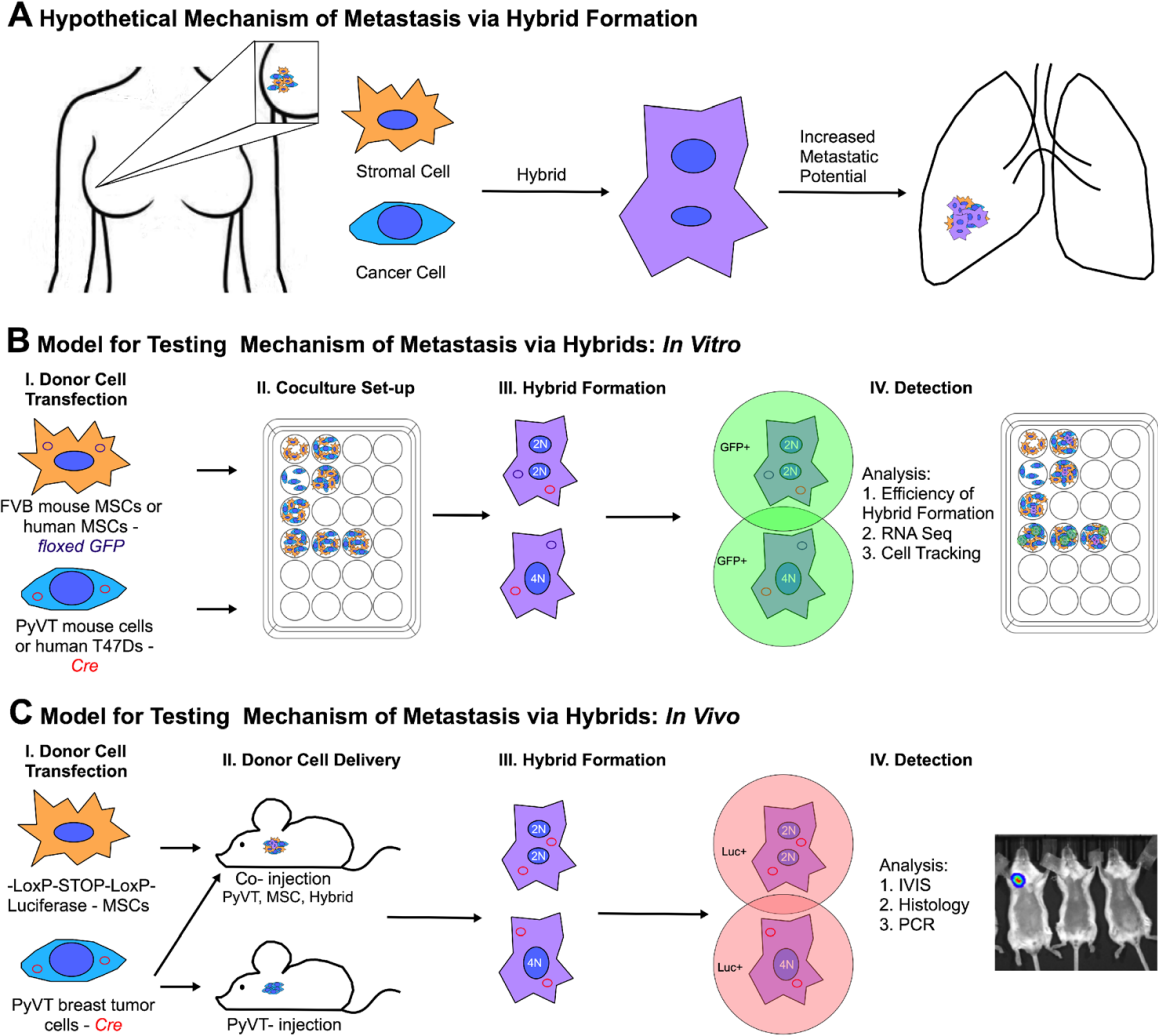
Schematic of the mechanism of metastasis and the experimental design. (a) Stromal cells and cancer cells form hybrids spontaneously *in vivo*; such hybrids contribute to metastatic mass. The hybrids are well-equipped to migrate out of the primary tumor and reestablish themselves at secondary sites. (b) Scheme to evaluate the metastatic potential of tumor-stroma hybrids *in vitro*. (c) Scheme to evaluate the metastatic potential of tumor-stroma cell hybrids *in vivo*.

Ninety percent of cancer-related deaths is due to secondary tumors or metastases, that form at sites far removed from the primary tumor. To successfully relocate in the body, a tumor cell must acquire transient properties that enable dissemination, followed by the reestablishment of the original primary phenotype at a distant site. Exactly how this is accomplished is yet unclear. One hypothesis suggests that a cancer cell acquires metastatic characteristics via accumulation of somatic mutations.^1,2^ However, a recent report compared the *entire genome* of a primary tumor cell with a corresponding metastatic tumor cell and found only two *de novo* mutations in the metastatic tumor; neither of the mutations were essential to the metastatic process.^3^ A more recent hypothesis suggests that a small population of cancer stem cells exists in a tumor capable of differentiation and reprogramming based on cues from the microenvironment.^4–7^ Though the cellular origin of cancer stem cells has been linked to both stem cells and differentiated cells, the natural mechanisms by which this unique cell type is generated are unclear.^8,9^ Here, we seek to test a third hypothesis (which may in fact explain the origin of cancer stem cells) that the exchange of the cellular material between tumor cells and stromal cells gives rise to hybrid cells capable of contributing to bona fide metastatic tumors [Fig. 1(a)].

The possibility that cell fusion gives rise to the metastatic phenotype was first proposed nearly a century ago by Aichel.^10^ Since then, transplantation studies have provided some support. For example, cells of a melanoma clone (wild type for tyrosinase, C/C) were implanted into BALB/c *nu/nu* mice (homozygous mutation for albino tyrosinase, c/c). After several weeks, massive pulmonary metastases developed. Cells of the metastatic tumors were cloned, and DNA analyses of the nucleotide sequences of exons 1 and 2 of the tyrosinase gene showed that most clones from the metastases had acquired the c allele (same as that of the *BALB/c* recipient) while maintaining the C allele. Thus, lung metastases were comprised primarily of host-tumor hybrids; interestingly, these hybrids expressed the same traits of enhanced motility and melanocyte stimulating hormone (MSH)/isobutylmethly xanthine (IBMX) responsiveness as *in vitro*-derived melanoma-macrophage hybrids.^11^ The first clinical confirmation of tumor cell fusion was found in myeloma patients in which more than 30% of osteoclast nuclei were found to be of tumor-cell origin.^12^ Other studies have shown the presence of donor genes in tumor cells of malignant tumors arising after allogeneic hematopoietic stem cell transplantation.^13–15^ Some conclude that fusion provides a means by which adherent cells acquire new qualities necessary to form metastases (i.e., enhanced motility and matrix degradation) under conditions conducive to hematopoietic survival and later resume tumor-like activities (i.e., rapid proliferation and cell-cell association) under conditions conducive to epithelial survival.

That fusion occurring with metastasis is now clear, but whether metastases are enabled or at least facilitated by fusion has only recently been tested. Zhou *et al.*^16^ induced hybrid formation between healthy rat epithelial cells artificially using poly(ethylene glycol). Hybrids were cloned and then injected into immunodeficient mice where only hybrids were able to generate tumors. Of these, some formed tumors with clear borders between muscle fibers (unlikely to metastasize), but others showed extensive invasiveness into muscle layers. Building on this important work and our own work showing breast tumor cells fuse *spontaneously* with mesenchymal stromal cells,^17^ we evaluate whether hybrids formed spontaneously *in vivo* contribute to bona fide metastatic tumors. To this end, we have developed an *in vivo* approach to trigger bioluminescence upon hybrid formation [Figs. 1(b) and 1(c)] and thus a means to determine if merging of the content of tumor cells with nearby cells occurs spontaneously in animals and, if so, whether hybrids of this type are more prevalent in the primary tumor or metastases. (Of note, we use the term “hybrid” throughout this work to reference cell-cell fusion and also the possibility of other modes of material transfer, namely, tunneling nanotube formation and exosome transfer.) We show that hybrids do in fact occur spontaneously *in vivo*, and in nearly all animals studied, a significantly higher number of hybrids are found in the metastases compared to the primary tumor, which supports the possibility that hybrids can emerge from the primary tumor and help create a new tumor at a distant site.

## RESULTS

### Tumor cell-stromal cell hybrids exhibit stochastic expression profiles that can preserve key functional pathways of each hybrid partner

**FIG. 2.**
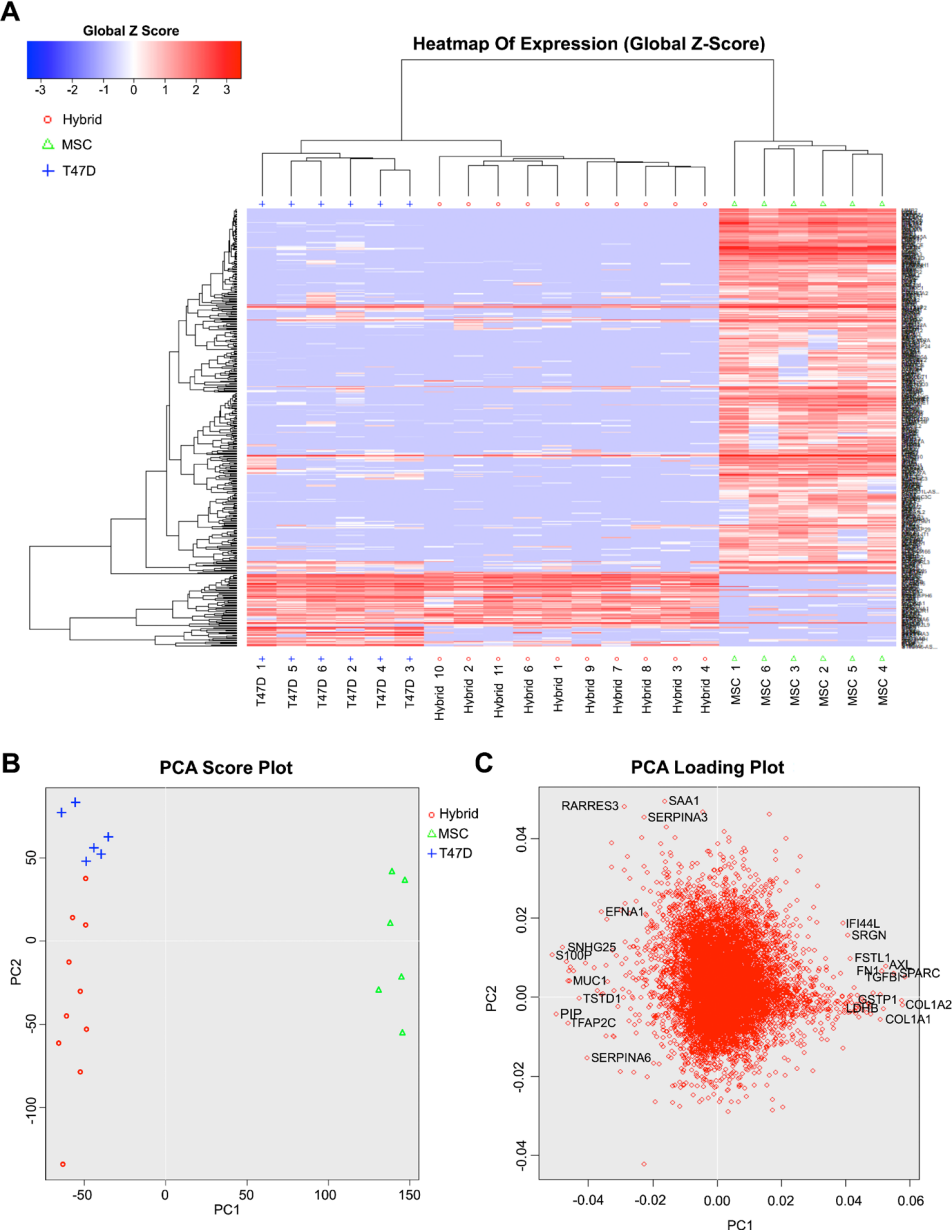
Hierarchical clustering (HC) and the principle component analysis (PCA) of all genes (with FPKM >1) of T47D-MSC hybrids and parental controls. (a) A global view of gene expression of hybrids (fusion 1–4, 6–11) and parental cells (T47D 1–6 and MSC 1–6). The global Z-score reflects the number of standard deviations away from the mean of expression of all genes in the display. Gene expression is shown in fragments per kilobase of exon per million fragments mapped (FPKM). (b) PCA score plot of hybrids and parental cells. (c) PCA loading plot showing the genes, which have the most substantive effect on component spread.

There are at least thirty-five reports of fusion between tumor cells and host cells.^18^ These include our recent work showing that human mesenchymal stromal cells (hMSC) are capable of spontaneous fusion with human breast tumor cells and that resultant fusion products acquire enhanced migratory^17^ and invasive^19^ capacity relative to unfused tumor cells. This is perhaps not surprising as stromal cells are capable of motility, chemotaxis, and matrix degradation/remodeling—all attribute that a tumor cell might require to undergo metastasis. We now seek to more comprehensively probe the metastatic potential of hybrids formed between these two cell types using single cell RNASeq. Specifically, hMSCs were transfected with a plasmid encoding floxed GFP; resultant populations were termed as FloxGFP_hMSC. FloxGFP_hMSC was plated at a density of 12 500 cells/cm^2^ on a gelatin coated 24 well plate. Twenty-four hours later, T47D breast cancer cells (human ductal breast epithelial tumor cells) were transfected with a plasmid encoding *Cre* recombinase; resultant populations were termed as *Cre*_T47D. *Cre*_T47D were plated at a density of 40 000 cells/cm^2^ on top of the FloxGFP_hMSCs. Under these conditions and 48 h after initiation of co-culture, we observe spontaneous hybrid formation at a rate of ∼1:1000 cells [supplementary material Fig. 1(A)]. At this same time point, co-cultures were harvested, and GFP^+^ cells, indicative of hybrids, were sorted using fluorescence activated cell sorting (FACS; supplementary material Fig. 1(A)]. Single cell RNASeq was performed on the hybrids (Fusion_n; n = 11 collected, Fusion_5 excluded due to poor quality messenger RNA (mRNA)) and corresponding parental cells (T47D_n; MSC_n; n = 6 of each). Hierarchical clustering (HC) and the principal component analysis (PCA) were conducted on all genes with Fragments Per Kilobase of transcript per Million mapped reads (FPKM) >1 for any sample [Figs. 2(a) and 2(b)]. HC and PCA plots showed control populations clustering according to the cell type and distinct from the hybrids. The hybrids also cluster with each other, and while the PCA distribution is highly spread, we note that the extent of heterogeneity is lower than that observed with artificially induced [i.e., using poly(ethylene glycol) or virus] fusion.^20^ The PCA loading plot shows a sampling of genes that contribute most substantially to the distance between individual cells within and between populations [Fig. 2(c)]. Interestingly, the genes that distinguish MSCs are related to extracellular matrix (ECM) remodeling (e.g., SRGN, serglycin proteoglycan; AXL, AXL receptor tyrosine kinase that binds ECM; SPARC, cysteine rich acidic matrix-associated protein; TGFb1, transforming growth factor beta 1 regulates ECM synthesis; and COLIA1/A2, collagen type I alpha 1 and alpha 2 chains), and a few genes that distinguish T47Ds are tumor suppressive in nature (e.g., RARRES3, retinoic acid receptor responder) and modulators of inflammation (e.g., SERPINA3, serpin family A member and protease inhibitor; SAA1, serum amyloid A1 expressed with inflammation). Meanwhile, hybrids were distinguished via genes associated with development and so perhaps more MSC-like (e.g., EFNA1, ephrin A1 tyrosine kinase heavily involved in neuronal development; TFAP2C transcription factor AP-2 gamma involved in the activation of several developmental genes) but also with genes associated with the tumor form and function (e.g., MUC1, mucin 1, the aberrant function of which is associated with carcinoma). But overall, both associative statistical analyses suggest the hybrids most resemble the tumor cells, at least at early time points following hybrid formation.

**FIG. 3.**
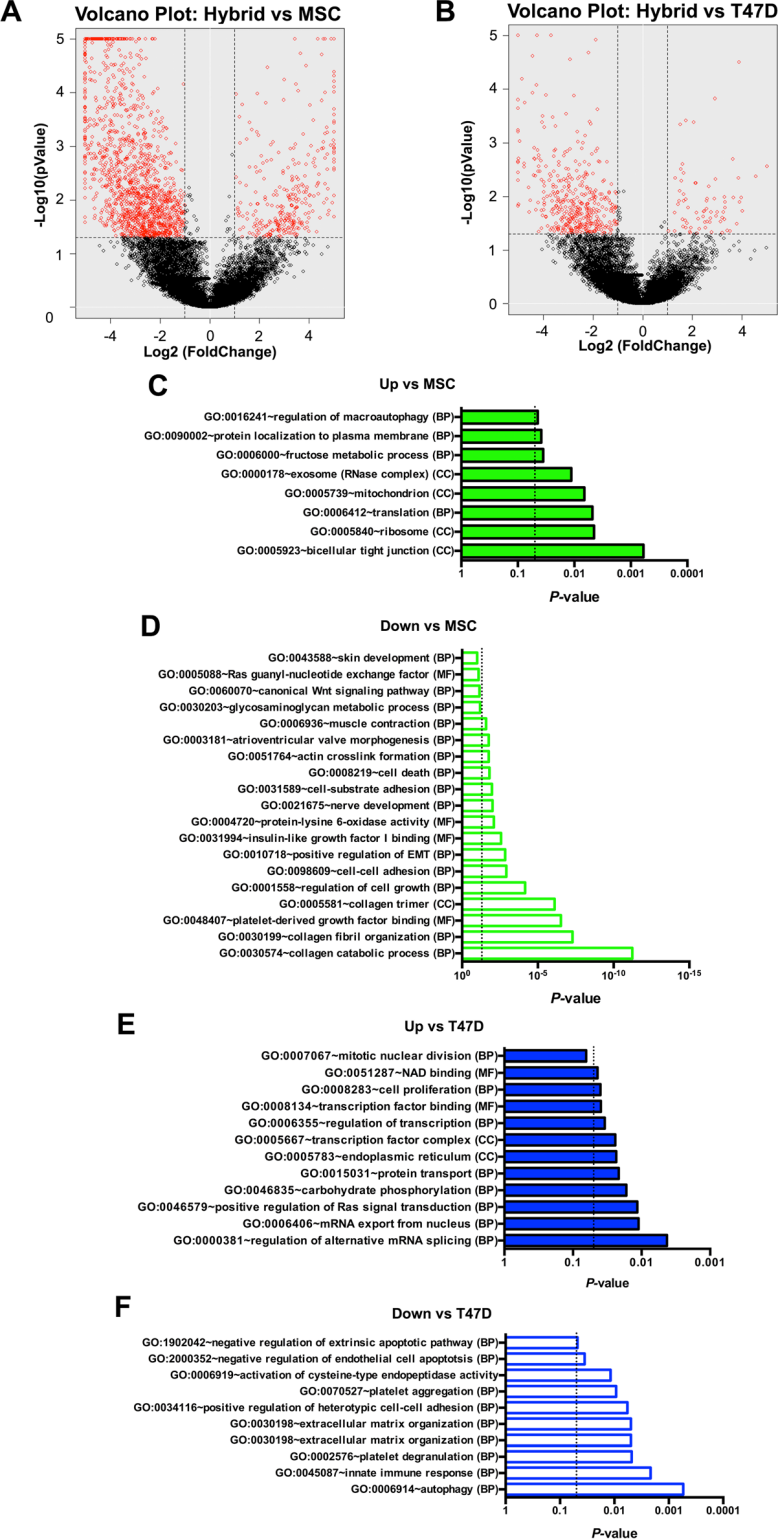
Volcano plots and gene ontology of hybrids with unique transcriptomes. (a) Volcano plot comparing hybrids with MSCs. (b) Volcano plot comparing hybrid fusion products with T47Ds. The P-value of functional annotation for differentially expressed genes identified by SCDE for ten hybrids (combined) with unique transcriptomes relative to either fusion partner. Increased FPKM values are denoted in closed green bars for MSCs (c) and closed blue bars for T47Ds (e). Decreased FPKM values are denoted in open green bars for MSCs (d) and open blue bars for T47Ds (f). The dashed line represents a *P*-value of 0.05. See also supplementary material Table 2.

To identify enriched, function-related gene groups and to isolate interacting proteins of the ten hybrids (combined) relative to parental cells (each group combined), we used the Database for Annotation, Visualization, and Integrated Discovery (DAVID) informatics resources. It should be noted that attributes unique to individual hybrids may be lost following the combination of gene profiles; however, there is not enough statistical power to be sure of the significance of comparisons between individual cells. Even so, genes significantly different between groups (i.e., hybrid, T47D, and hMSC genes) were identified using the Single Cell Differential Expression (SCDE) toolkit and separated according to increased or decreased FPKM values relative to each parental comparator and displayed as red circles in volcano plots of Figs. 3(a) and 3(b).^21^ These genes were then imported to the DAVID resource, and pathways corresponding to the most prominent, nonduplicating, gene ontology (GO) TERMS (BP, biological process; CC, cellular component; and MF, molecular function) were amassed and displayed as bar charts [Figs. 3(c)–3(f); the complete output can be found in the supplementary material, Table 2]. We comment here exclusively on genes that are increased relative to parental controls as these are more likely to represent maintenance or gain of the function, whereas genes that decrease in value may do so only as a function of transcript dilution that occurs as a function of the merging of the cytoplasmic material. We find that relative to hMSC, hybrids show an increased expression of mitochondrial elements suggesting that the hybrids have increased energetic demands. They also show an increased cell junction assembly supporting the ability of hybrids to contribute or establish tumors whether at the primary or distant sites. Relative to T47D, hybrids show an increased expression of mRNA processing elements, endoplasmic reticulum maintenance, and protein transport, suggesting increased protein synthesis similar to hMSC cells. Cell proliferation pathways were also increased which is somewhat unexpected as it might be instead predicted to increase relative to hMSC and perhaps not to the tumor parent. Finally, mitogen-activated protein kinase (MAPK)/RAS signaling was increased, a pathway associated with engagement of proteins of the extracellular space and polymerization of the actin cytoskeleton in association with adhesion and/or migration.

### Enhanced migratory capacity of murine breast tumor-stromal cell hybrids in vitro

Our previous work^17,19^ and that utilizing RNASeq (above) was conducted with human cell types, but here we transition to mouse cells so that outcomes might be associated with *in vivo* mouse studies [Figs. 1(b) and 1(c)]. In particular, murine mammary tumor cells were isolated from spontaneously formed tumors of the fat pad of female mice, hereafter termed PyVT cells. These mice express the Polyoma Virus middle T antigen under the direction of the mouse mammary tumor virus promoter/enhancer and, therefore, develop palpable mammary tumors, which metastasize to the lung. In parallel, murine bone marrow-derived mesenchymal stromal cells were isolated from FVB.129S6(B6)-*Gt(ROSA)26Sor^tm^*^1*(Luc)Kael*^/J, hereafter termed FloxLuc_mMSC (supplementary material, Fig. 2, the characterization of friend leukemia virus B (FVB) strain wild type mMSCs). These mice contain the firefly luciferase (*luc*) gene inserted into the *Gt(ROSA)26Sor* locus such that expression of the luciferase gene is blocked by a loxP-flanked STOP fragment placed between the *luc* sequence and the *Gt(ROSA)26Sor* promoter. These mice were chosen such that *in vivo* hybrid formation with a cell expressing *Cre* recombinase might be detected in live mice via luminescence of substrate-bound luciferase. For *in vitro* studies, detection of hybrids cannot be easily discerned at the level of the single cell using luminescence, and so, stromal cells of FVB wild type mice were transfected with a plasmid encoding floxed GFP, hereafter termed FloxGFP_mMSC. A day later, PyVT cells were transfected with a plasmid encoding *Cre* recombinase, hereafter termed Cre_PyVT in the text and PyVT in the figures. These two cell types were co-cultured such that approximately 50% of the surface area was covered by each cell type and also such that the confluence was 90%–100% on day one to ensure close cellular apposition. The frequency of hybrid formation after 48 hours of initiation of co-culture was 1.07% ± 0.44% or approximately 1/95 cells, on the same order of magnitude as that observed for spontaneous fusion of similar human cell types *in vitro.*^17^ This number may be inflated if the hybrids proliferate, but it could also be an underestimate as the transfection efficiency is less than 100% for plasmids of both fusion partners. The frequency of *nuclear* fusion, as assessed by fluorescence *in situ* hybridization (FISH) for sex chromosomes, was slightly less at 0.85% ± 0.30% or approximately 1/120 cells (supplementary material, Fig. 3) and represents the fraction that underwent bona fide cell fusion (approximately 79%). The remaining hybrids (21%) may have transferred material as a consequence of cell fusion but not nuclear fusion, exosome transfer, tunneling nanotube formation, or other yet undescribed modality.

**FIG. 4.**
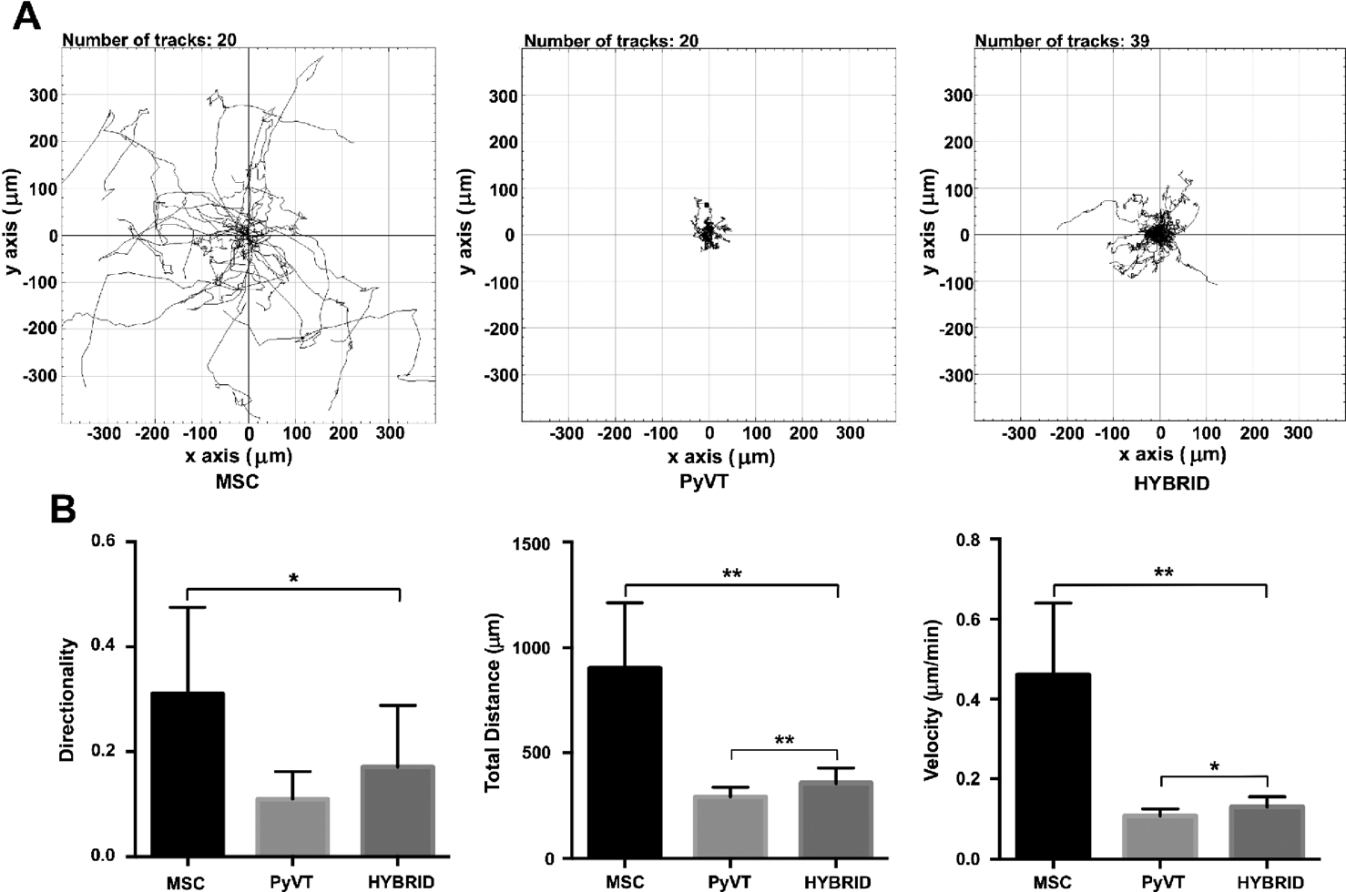
Hybrid cells show increased migratory capability. Migration of hybrid cells was compared to that of parent cells of single cell cultures using time-lapse microscopy. (a) Representation of motility patterns of FloxGFP_mMSCs, Cre_PyVTs, and hybrids. (b) Directionality of motion of cells, the total accumulated distance, and velocity for each group. Each value (mean ± SD) reflecting the average of 2 separate experiments, each with at least two different wells, including the analysis of at least 9 cells per group per well. Mann-Whitney: *P ≤ 0.001 and **P ≤ 0.0001. PyVT, Cre_PyVT; MSC, FloxGFP_mMSC.

We previously observed that upregulation of pathways associated with MAPK/RAS in hybrids translates to enhanced migratory capacity in the case of *human* tumor cell-stromal cell hybrids.^17^ To determine whether the same is true for *mouse* tumor cell-stromal cell hybrids, we utilized time-lapse microscopy to analyze the migratory capability of hybrids derived from the fusion of Cre_PyVT and FloxGFP_mMSC cells. Time-lapse imaging was initiated 4 h after co-culture seeding, and images were taken every 30 min for 72 h (Fig. 4). PyVT cells (of single cell culture) show little movement with an average total distance and velocity of 292.9 ± 44.8 *μ*m and 0.11 ± 0.02 *μ*m/min, respectively, while mMSCs (of a single cell culture) were highly motile with an average total distance and velocity of 901.9 ± 311.4 *μ*m and 0.18 ± 0.003 *μ*m/min, respectively. mMSCs additionally showed directed travel patterns not seen within the PyVT population with a directionality value of 0.31 ± 0.17 as compared to PyVT at 0.11 ± 0.05. The hybrids (of co-cultures) acquired a modest but a significant increase in the total distance and velocity as compared to the PyVT parent cells (356.5 ± 71.3 *μ*m and 0.13 ± 0.03 *μ*m/min, respectively) and showed a directionality value in between both parent cells at 0.17 ± 0.12 though not significantly different than PyVT cells alone. Similar trends were observed when hybrids were compared to parental cells of the co-culture, thus eliminating the possibility that soluble factors of the co-culture alone could account for changes in the migratory behavior of the hybrids (supplementary material, Fig. 4).

### PyVT cells form hybrids spontaneously with cells of the tumor microenvironment in vivo

**FIG. 5.**
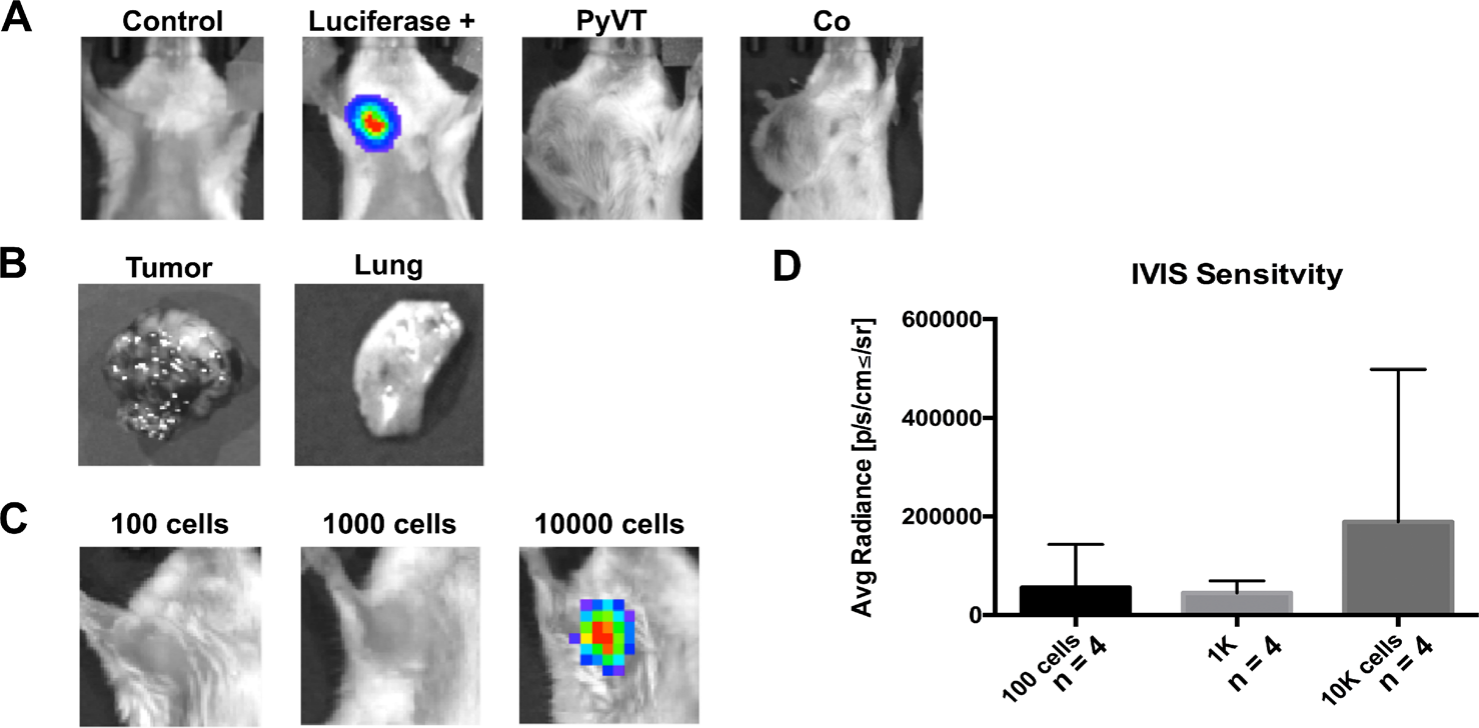
IVIS sensitivity limits hybrid detection *in vivo*. (a) IVIS spectrum images taken post-injection of control (no cells), luciferase + (MCF7-luc), PyVT, and Co cell cultures into the fat pad. (b) Primary tumor and lungs did not express a luciferase signal when imaged *ex vivo*. (c) IVIS sensitivity was tested by injecting different cell numbers of MCF7-luc into the fat pad to determine the level of cells needed to obtain a signal (d). PyVT, Cre_PyVT; Co, PyVT-MSC co-culture; and Luciferase +, MCF7 cells that constitutively express luciferase.

**FIG. 6.**
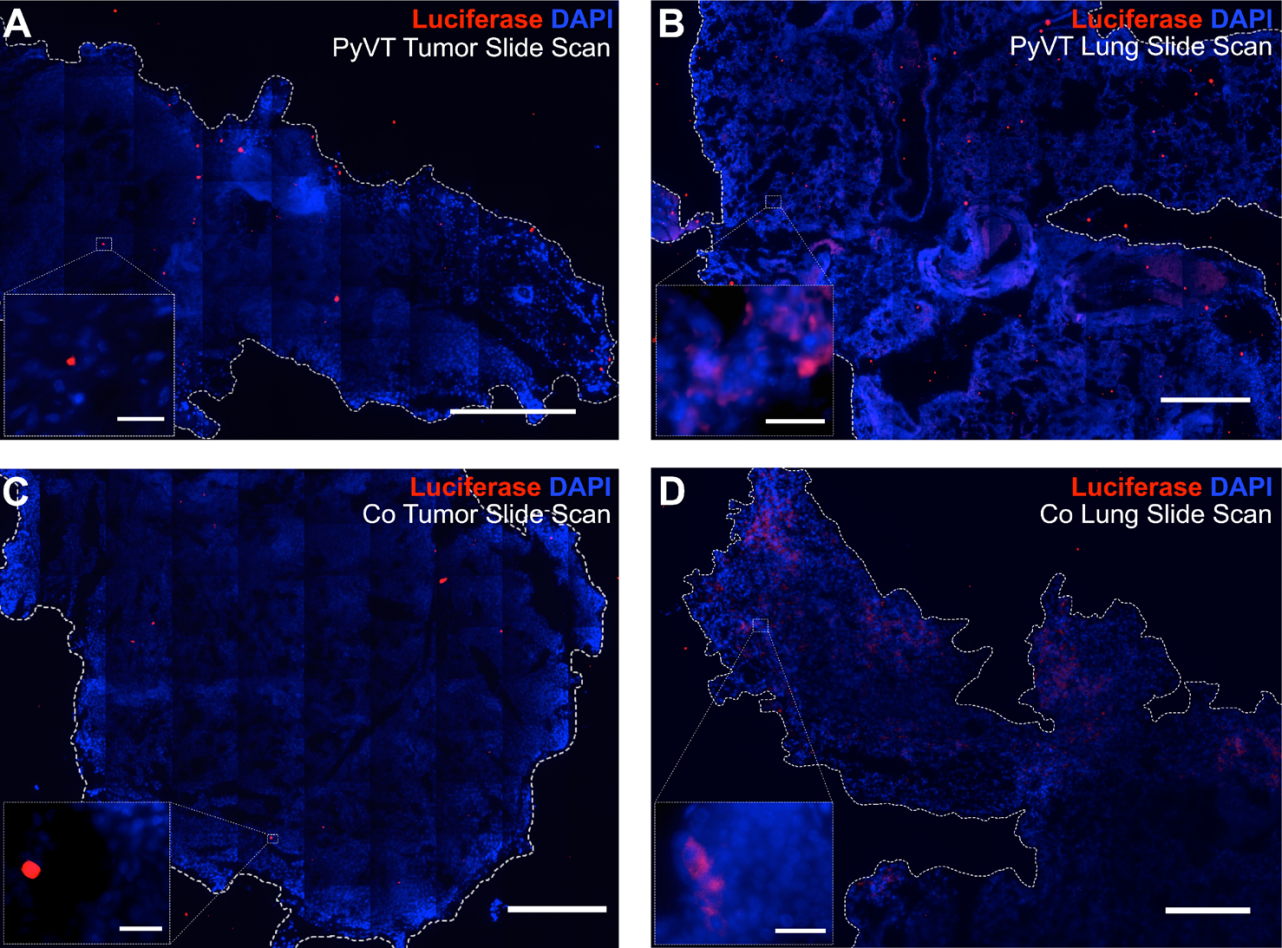
Immunofluorescence imaging of luciferase protein as a reporter of hybrid formation. Entire primary tumor and lung sections were imaged via tile scanning, and each image of the scan was carefully analyzed to confirm or refute positive staining for luciferase. The luciferase signal was considered a positive signal if it was above background levels associated with negative controls and corresponded to the cytoplasm of a cell with a nucleus. Rare luciferase-positive cells were detected in the primary tumors. Most red signal was not in the cytoplasm of cells associated with nuclei and, therefore, considered nonspecific [insets (a), (c)]. The lungs containing metastases on the other hand [(b), (d)] contained a large number of bona fide luciferase-positive cells corresponding to fusion products. Scale bars on slide scans = 100 *μ*m. Scale bars on 40× inset = 25 *μ*m.

**FIG. 7.**
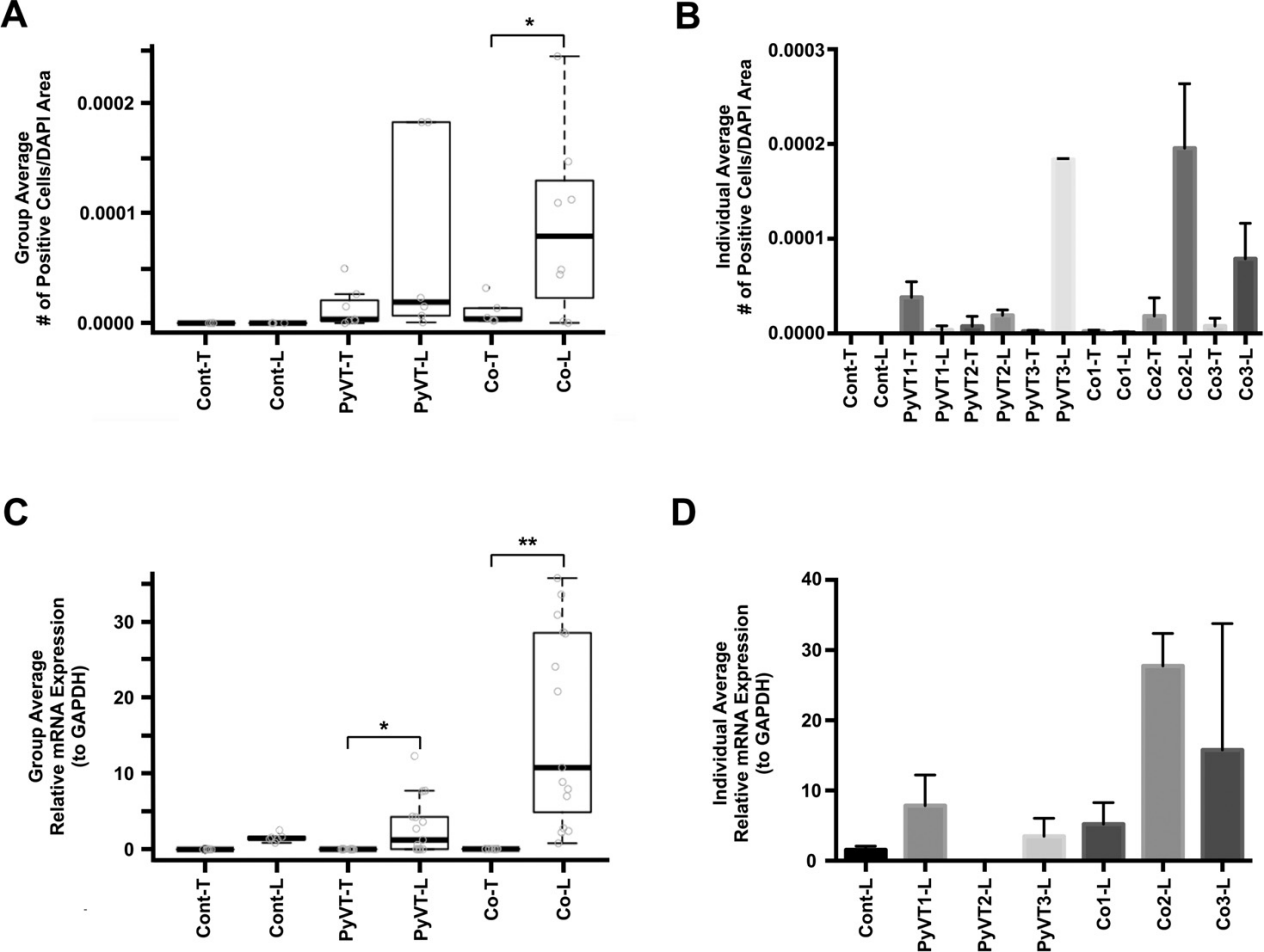
Quantification of luciferase expression in primary tumors and lung metastases. Individual images from slide scans (Fig. 6) were evaluated by counting the number of positive cells/DAPI area. (a) Average number of positive cells per DAPI area per experimental group, n ≥ 3 slides from n ≥ 3 animals per experimental group. Student's t test comparing tumor to lung metastases for each type of cell injection, mean ± SD, *P < 0.05. (b) Average number of positive cells per DAPI area per animal, n ≥ 3 slides with 50–400 images per slide per animal. The expression of firefly luciferase was measured using quantitative reverse transcription polymerase chain reaction (qRT-PCR) and normalized to the expression of glyceraldehyde 3-phosphate dehydrogenase (GAPDH). (c) Average relative expression of luciferase mRNA per experimental group, n ≥ 3 animals per experimental group. Mann-Whitney comparing tumor to lung metastases for each type of cell injection, mean ± SD. *P < 0.01 and **P < 0.001. (d) Average relative expression of luciferase mRNA per animal, n ≥ 3 technical replicates. Only results from lung tissues are shown as results from individual tumors did not exceed background. Cont and PyVT cells were not transfected with *Cre* recombinase and injected in the fat pad of FVB.129S6(B6)-*Gt(ROSA)26Sor^tm^*^1*(Luc)Kael*^/J mice and injected with luciferin prior to tissue harvest; PyVT and Cre_PyVT injected in the fat pad of FVB.129S6(B6)-*Gt(ROSA)26Sor^tm^*^1*(Luc)Kael*^/J mice and injected with luciferin prior to tissue harvest; Co and Cre_PyVT-FloxLuc_mMSC co-cultures were injected in the fat pad of FVB.129S6(B6)-*Gt(ROSA)26Sor^tm^*^1*(Luc)Kael*^/J mice and injected with luciferin prior to tissue harvest; T, Primary Tumor; L, Lung Metastasis; number labels indicate the mouse identifier.

To evaluate whether tumor cell hybrids (formed with stromal cells or other component of the tumor environment) contribute to metastases, we first determined whether hybrid formation can occur spontaneously *in vivo.* To this end, 1000 Cre_PyVT cells were injected into the fat pad of FVB.129S6(B6)-*Gt(ROSA)26Sor^tm^*^1*(Luc)Kael*^/J mice. In this way, hybrids should express luciferase where expression of luciferase is not dependent on continued expression of *Cre* once the initial excision has been made. At days 1, 3, 7, 14, 28, and monthly until the primary tumor was 2 cm in diameter, animals were injected with luciferin and imaged using an IVIS imaging system. When the tumor was approaching or had reached 2 cm in diameter, continued life would be uncomfortable for the animals. Unfortunately, we were never able to discern a reliable luciferase signal either in the intact animal or upon excision of the tumor or metastases at any time point [Figs. 5(a) and 5(b)]. The level of reliable detection with this modality and assuming that all positive cells are in close proximity to each other is 10 000 cells in the fat pad [Figs. 5(c) and 5(d), bolus injection]. To probe with a finer resolution, especially since hybrids may not be in close proximity to each other in tissues, tumors were stained with an anti-luciferase antibody (supplementary material, Fig. 5, staining of the control cell line and control mouse tissues) and assessed via quantitative reverse transcription polymerase chain reaction (qRT-PCR) for luciferase mRNA. Of thirteen mice injected, three developed macroscale metastases in the lung (23%). We probed the primary tumors of the three animals that developed metastases. Of the tumors analyzed, only the three primary tumors with metastases exhibited discernable but very sparse expression of luciferase with an estimated frequency of 1 in 10 000 cells [Figs. 5(a) and 6(a)]. These same tumors were negative for luciferase mRNA [Fig. 7(c)]. It should be noted that the number of hybrids putatively detected represents the culmination of several months of activity, which could include death or migration (and thus an underestimate of the initial frequency of fusion) or proliferation (and thus an overestimate of the initial frequency of fusion).

### PyVT hybrids are more prevalent in lung metastases than the primary mammary tumor

**FIG. 8.**
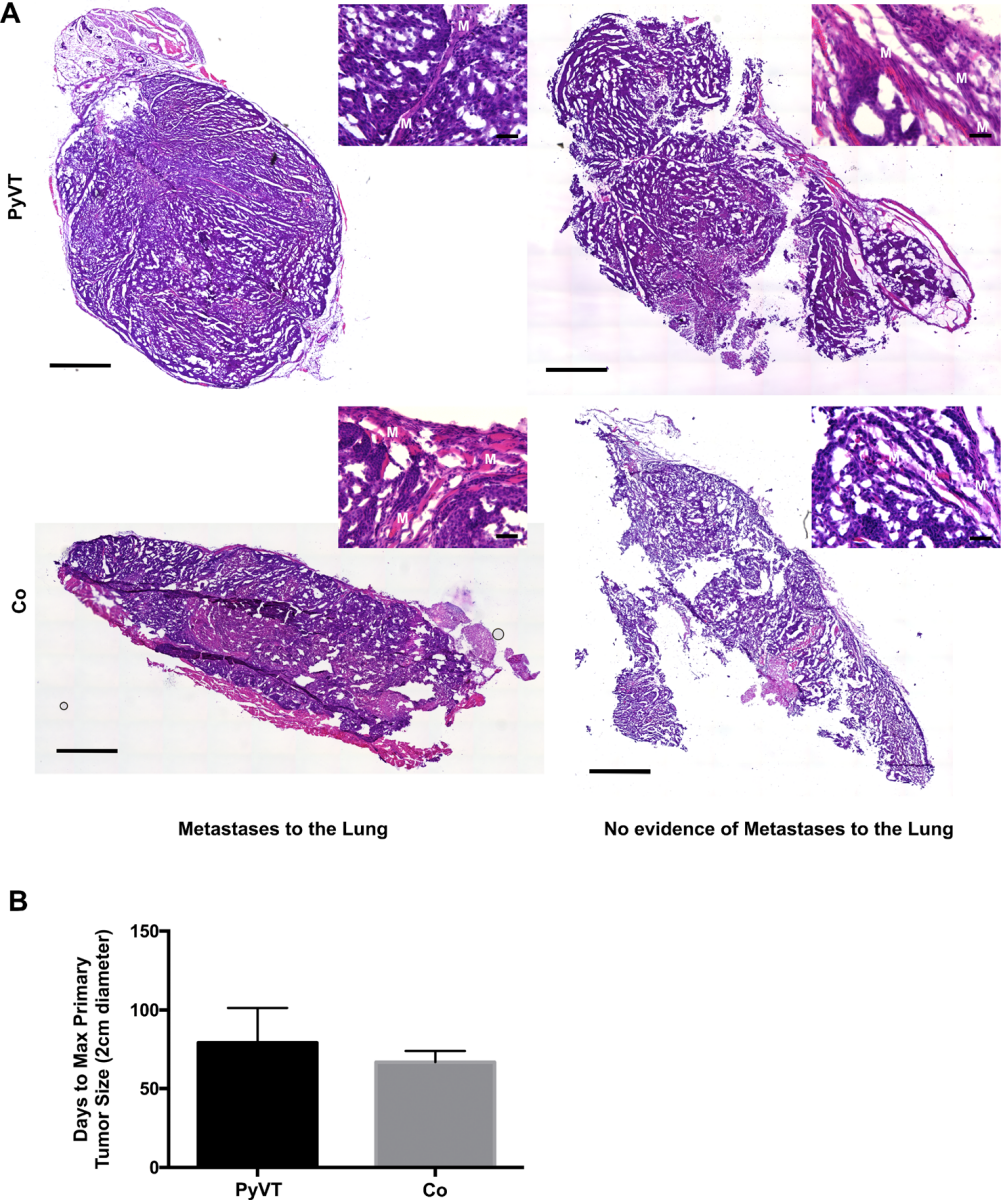
Assessment of the tumor invasive morphology and time to endpoint. (a) Tumors were removed from mice and stained with hematoxylin and eosin. All tumors showed signs of invasiveness whether or not visible metastases were detected in the lung. Muscle is labeled with a white M. Slide scan: scale bar 1000 *μ*m. 20× inset: scale bar 50 *μ*m. (b) Average time to maximum primary tumor size (2 cm) was lower in co-culture (Co) injected mice relative to PyVT injected mice. PyVT, Cre_PyVT; Co, Cre_PyVT-FloxLuc_mMSC co-culture cells that included fusion products.

To determine whether tumor cell hybrids could populate metastases of mammary tumors, we tracked the lung metastases of the three animals with spontaneous hybrid formation in the primary tumor using IVIS imaging. Again, the positive signal was not observed via bioluminescent imaging either in the intact animal or in the excised lung at all time points up to sacrifice (at 2 cm diameter primary tumor, typically 9.5 weeks). Given the lack of the luminescent signals, indicative of hybrid formation, we analyzed the tumors for evidence of the invasive morphology including infiltration to the muscle layers, suspecting that those tumors with metastases would exhibit a primary tumor with more invasive morphology than those without metastases. However, we found that all primary tumors appeared primed for invasion consistent with the history of the PyVT model^22,23^ (Fig. 8). Then, and as with the primary tumors, the metastases of all three animals were probed with an anti-luciferase antibody and were found to contain luciferase-positive cells via immunofluorescence [Fig. 6(b)]. Importantly, the number of hybrids detected per 4′,6-diamidino-2-phenylindole (DAPI) area of the metastases was higher than the number of hybrids detected per DAPI area of the primary tumor (Fig. 7). In addition, luciferase mRNA could be reliably detected in the metastases at a level statistically higher than that of the tumor (Fig. 7). In an attempt to bolster the number of hybrids housed in the primary tumor, co-cultures of Cre_PyVT and FloxLuc_mMSC at a ratio of 1:1 (termed Co; containing the same number of Cre_PyVT as the single cell type injection but with matched numbers of mMSCs) containing approximately 1% of Cre_PyVT:FloxLuc-mMSC hybrids were injected to the fat pad. We found that the primary and secondary tumors contained similar numbers of hybrids per DAPI area compared to the Cre_PyVT alone injections, and importantly, that the number of hybrids per DAPI area in the metastases was significantly higher than that of the primary tumor. The time to primary tumor endpoint was shorter on an average with Co injection compared to Cre_PyVT only injection though not statistically significant [66.8 ± 7.1 and 79.2 ± 22.1 days, respectively, Fig. 8(b)]. In addition, there is a slight increase in the fraction of Co injected mice to develop metastases (27%) relative to the Cre_PyVT injection alone. This suggests that injection of co-cultures containing hybrids might accelerate the formation of the primary tumor but does not appear to alter the metastatic morphology of the primary tumor.

## DISCUSSION

This report is the first, to our knowledge, to show that tumor cell hybrids formed spontaneously *in vivo* contribute more substantially to metastases than to primary tumors adding credence to the possibility that hybrid formation enables metastasis. There are many reports of hybrid formation between tumor cells and host cells, and many previous reports implicate bone marrow-derived cells of myeloid lineage as host fusion partners (reviewed in Refs. 24 and 25). In addition, a number of *in vitro* and *in vivo* studies have now shown that hybrids generated from the fusion between primary tumor cells and cells of hematopoietic lineage can result in metastatic cells.^13,26–30^ To highlight one of the several recent examples, Li *et al.* showed that human hepatocellular carcinoma cells with a low metastatic potential exhibit a significantly increased metastatic potential following fusion with mesenchymal/multipotent stem/stromal cells (MSCs) *in vitro* and in xenograft studies.^31^ Of note for these studies, fusion occurred first *in vitro* causing some to question the relevance for patient populations. This debate continues despite the discovery in cancer patients of circulating tumor cells expressing both carcinoma and leucocyte cell markers supporting hybrid formation between bone marrow-derived cells and tumor cells.^12–14^ Interestingly, a study further demonstrated that macrophage-melanoma hybrids found in the peripheral blood of patients with cutaneous melanomas produced metastatic lesions at distant sites when transplanted subcutaneously in nude mice.^32^ Those hybrids in patients' peripheral blood might have been on their way to distant sites to develop metastases. In further support, two recent reports used short tandem repeat length-polymorphism and forensic genetic techniques to show that metastatic melanoma lesions in two patients arose from the fusion between bone marrow-derived cells the patients received as a transplant and tumor cells.^15,33^ It has been suggested that after fusion between cancer cells and bone marrow-derived cells, the hybrids undergo epithelial to mesenchymal transition (EMT) and acquire stem cell-like properties that enhanced their tumorigenicity and ability to metastasize. But as time goes by, those hybrids reacquire epithelia-like morphology a process termed mesenchymal-epithelial transition or MET.^34^

Although evidence of hybrid formation in metastases is mounting,^14,15,33^ spontaneous tumor cell hybrid formation *in vivo* itself has never been shown to initiate or contribute to metastasis. We assessed in this study whether tumor cell hybrids formed spontaneously *in vivo* can contribute to bona fide metastatic tumors. We injected tumor cells expressing Cre or co-culture of tumors cells expressing Cre and mMSCs expressing FloxLuc into the fat pad of FVB.129S6(B6)-*Gt(ROSA)26Sor^tm^*^1*(Luc)Kael*^/J mice and recorded luciferase protein expression as one indicator of hybrid formation. These results were supported by qRT-PCR for luciferase gene expression in the same tissues. Of the three possible routes of hybrid formation, including cell-cell fusion, exosome-mediated protein transfer,^35,36^ or tunneling nanotube formation,^37,38^ we suspect that cell-cell fusion dominates. Specifically, our *in vitro* studies showed a high fraction of multinucleated hybrids [supplementary material, Fig. 3(a)], and FISH staining for sex chromosomes showed several cells with multiple and often odd numbers of X and Y chromosomes contributed by MSC (isolated from male mouse) and tumor cell (isolated from female mouse), suggesting nuclear fusion within hybrids [supplementary material, Fig. 3(b)]. To our knowledge, exosome transfer and tunneling nanotube formation are not known to support transfer of whole chromosomes or nuclei. In addition, we found a significantly higher number of cancer cell hybrids in the metastases compared to the number in the primary tumor, which supports the possibility that fusion products can emerge from the primary tumor and proliferate to help create a new tumor at a distant site. Corroborating this view, Lazova *et al.* showed that a melanoma metastasis in a patient who had received bone marrow transplant must have resulted from a clone through a single fusion-hybridization event between a bone marrow-derived cell and a tumor cell.^33^

Spontaneous hybrid formation between cancer cells and other cells seems to occur at a relatively low frequency in the primary tumor and the fraction of hybrids detected in metastases, while higher than in the primary tumor, is still less than a few percent of the total tissue mass. A reasonable question is whether this low frequency is meaningful for the formation of metastases. The answer is yet uncertain, but the possible import is still quite high in our view. Indeed, Al-Hajj *et al.* showed nearly 15 years ago that as few as 100 cells equipped with unique properties can give rise to a tumor,^37^ and more recently, 1 tumor initiating cell has been shown to give rise to a tumor.^38^ If one cell can give rise to a primary tumor, it stands to a reason that one cell can give rise to a metastasis; therefore, in our view, the frequency of hybrid formation does not limit the potential impact of this process. Why then, are not all, or at least a larger fraction of the cells of metastases hybrids? One reason could be technical in nature and attributed in part to the fact that the transfection efficiency of the transplanted cells with the *Cre* expression plasmid is not 100%. Therefore, there are other hybrids that we do not detect, and it could be these that have a competitive advantage in the end over other hybrids that we can detect. In addition, it could be that the hybrids quickly lose/recycle extra genetic and cytoplasmic materials, thereby losing the reporter. From a biological standpoint, it could be that hybrids form the initial “tracks” out of the primary tumor and that once created, other tumor cells can more easily follow. Future work to assess the position of hybrids relative to extracellular matrix proteins and blood vessels soon after tumor formation and at prescribed intervals thereafter would help to discern whether this hypothesis holds. Along those lines, it could be that the hybrids create the “correct” local microenvironment for tumor escape to take place. Testing of this possibility could include evaluation of soluble factors secreted by hybrids.

In the end, the most compelling means to establish whether hybrids facilitate metastases is to inhibit and then recover hybrid formation. Unfortunately, while hybrid formation is essential in developmental,^39^ physiological,^40^ and pathological^41^ processes in eukaryotes, the underlying mechanisms regulating hybrid formation are still being discovered. Adhesion complexes and cytoskeletal structures together with fusogens have been described in cell-cell fusion events during development and fertilization (reviewed in Ref. 42). However, studies to unravel the molecular mechanisms and signaling pathways governing *cancer cell hybrid formation* are in their infancy.^18^ Delineating those mechanisms will help define the role of hybrid formation in metastases. If hybrid formation is found to be a driver of metastases, understanding mechanisms of hybrid formation could also spur development of a new class of drugs for cancer treatment that will inhibit cancer cell hybrid formation and, therefore, may prevent metastatic spread.

The means by which hybrid formation facilitates metastases may extend beyond the capacity to preserve functional pathways of two discrete cell types. Hybrids also, by default, generate heterogeneous and unique cell types. Importantly, hybrid partners of tumor cells *in vivo* are not yet known and might be diverse, adding to the heterogeneity. This heterogeneity could represent the driving force not only for the development of metastases but also in the emergence of resistance of tumor cells to treatment. A recent study showed that the merging of breast cancer cells (MCF7) and macrophages gives rise to hybrids resistant to radiation and exhibiting enhanced DNA-repair capacity.^43^ A different study demonstrated that hybrid clones derived from the same parental line could initiate, in immunodeficient mice, tumors exhibiting distinct rates of growth and histology as well as different degrees of invasiveness.^16^ It would, therefore, be of benefit for future work to probe more closely the expression profile of hybrids, like the RNAseq studies described here, that may contribute to the capacity to avoid specific therapeutics. In terms of other hybrid partners, our studies indicate MSCs spontaneously fuse with tumor cells and could be the hybrid partner *in vivo.* This possibility is supported by the fact that MSCs are localized to many tumor types,^16^ including breast,^44^ and at least a fraction of these appear to have originated from the bone marrow.^45,46^ Future studies, perhaps by coupling expression of the floxed reporter cassette to a cell-specific promoter, will help confirm putative partners and identify other partners that can contribute to the formation of tumor hybrids and confer associated tumor heterogeneity.

## METHODS

### Cell culture

Ethics approval for all *in vitro* studies was obtained from the University of Minnesota, Institutional Biosafety Committee (Protocol No. 1306-30681H). T47D cells were obtained from ATCC (HTB-133 FZ, ATCC) and not passaged for more than 6 months (approximately 25 passages) and were maintained in Roswell Park Memorial Institute Medium (RPMI 1640) with 10% of fetal bovine serum (FBS) (ES-009-B, EMD Millipore, Billerica, MA) and 1% of penicillin/streptomycin (P/S) (17-602E, Lonza, Basel, Switzerland). Human MSCs were derived from human embryonic stem cells and were fully characterized by the laboratory of Dr. Peiman Hematti^47^ (University of Wisconsin-Madison). hMSCs were maintained in α-MEM (Minimum Essential Medium Alpha Medium) (12000-022, Gibco by Thermo Fisher, Waltham, MA), 10% FBS (ES-009-B, EMD Millipore), 1% Non-Essential Amino Acids (NEAA) (11140-050, Gibco), 1% l-glutamine (250300-81, Thermo Fisher, Waltham, MA), and 1% P/S. Primary tumors were removed from MMTV-PyVT transgenic mice [002374-FVB/N-Tg(MMTV-PyVT)634Mul/J, Jackson Laboratory] and rinsed in a wash buffer [DMEM/F12 (1:1) (11320-033, Gibco), 1% FBS (ES-009-B, EMD Millipore), and 50 *μ*g/mL l/S] before mincing in a sterile petri dish with a scalpel for approximately 8 min or until paste-like consistency with few obvious chunks. The tissue was placed into a 50 ml conical tube with 25 ml of filtered collagenase digestion buffer [DMEM/F12 (1:1), 2 mg/ml of collagenase A (11088785103, Roche), and 100 U/ml of P/S] and incubated at 37 °C for 3 h on a rocker at 120 rpm. The tissue in the buffer was pipetted every 15 min until the end of the incubation period. The cells were centrifuged for 5 min at 1500 rpm, and the supernatant was removed. The cell pellets were washed three times with wash buffer for 5 min at 1500 rpm and then washed twice with wash buffer for 5 min at 800 rpm. The cells were counted and resuspended in the filtered plating medium [F12/DMEM and 5 *μ*g/insulin (11376497001, Sigma-Aldrich, St. Louis, MO), 1 *μ*g/ml hydrocortisone (H0888-5G, Sigma-Aldrich), 5 *μ*g/ml epithelial growth factor (EGF) (AF-100-15-1-MG, PeproTech, Rocky Hill, NJ), 50 *μ*g/ml gentamicin (15710-064, Gibco), and 100 U/ml P/S]. The cells were plated at 2.5 × 10^5^ per cm^2^ on dishes that had been coated for 1 h with filtered serum fetuin [F12/DMEM, 20% FBS, and 2 mg/ml fetuin (53385-1G, Sigma)]. The cells were allowed to attach for two days and then ongoing culture utilized the growth medium (plating medium + 10% FBS). For positive controls, MCF7 breast cancer cells constitutively expressing firefly luciferase (MCF7-luc, Xenogen) were maintained in DMEM/F12 with 10% of FBS.

Mouse mesenchymal stem cells (mMSCs) were isolated from 005125-FVB.129S6(B6)-*Gt(ROSA)26Sor^tm^*^1*(Luc)Kael*^/J and 001800-FVB/NJ mice (Jackson Laboratory). Mice (between 6 and 12 weeks old) were euthanized via CO_2_ exposure followed immediately by hind limb removal. Muscles were removed while being careful to avoid damage to the bone. The ends of the bones were cut with scissors and the diphysis flushed with α-MEM containing 20% FBS. The flushed media and cells were spun down at 300 g for 5 min. Cells of the bones from one leg were seeded on a gelatin coated well of a 6-well plate; numbers at this point in the process are difficult to determine accurately. Cells were maintained in α-MEM with 20% FBS, 1% l-glutamine, 100 U/ml P/S, and 1% NEAA for 3 days, with the first media change after 24 h. Cells were then maintained in α-MEM with 10% FBS, 100 U/ml P/S, and NEAA (mMSC media) and passaged when cells reached no more than 80% confluence. Cells were only used between passage four and seven.

### *In vitro* co-culture of tumor and stromal cells

Co-culture systems were optimized to promote the highest rates of spontaneous cell fusion. It was found that initiating cocultures by layering the T47Ds on the hMSCs on the 2nd day gave maximum number of fused cells. Moreover, electroporation yielded better results as opposed to transfection with Fugene (data not shown). Human co-culture were set up in 6-well plates to allow for using these cultures in future studies using the hMSC media. hMSCs were detached from plates, spun down and resuspended at 5000 cells/*μ*l in R buffer (NEON Transfection Kit, Thermo Scientific). hMSCs were electroporated using the following electroporation protocol: 1400 mV with 8 *μ*g/10^6^ cells of pBS185 CMV-Cre plasmid (Addgene) and plated at 500 000 cells per well. On day two, the media was changed, and T47Ds were detached from plates, spun down, and resuspended at 10 000 cells/*μ*l in R buffer. T47Ds were electroporated using the following electroporation protocol: 1700 mV using 8 *μ*g/10^6^ cells of the floxed GFP plasmid (pCALNL-GFP, addgene) and plated at 1 × 10^6^ cells per well on top of the hMSCs. The media was again changed after 24 h and every 48 h thereafter. Fusion products were detected 48–72 h later.

For mouse studies, mMSCs isolated from bone marrow of FVB/NJ base mice were used for *in vitro* experiments. mMSCs were detached from plates, spun down, and resuspended at 5000 cells/*μ*l in R buffer. The floxed GFP plasmid (pCALNL-GFP, Addgene) was added at 8 *μ*g/10^6^ cells to the buffer, and FVB-MSCs were electroporated using the following electroporation protocol: 1400 V, 20 ms, and 1 pulse. FloxGFP_mMSCs were plated at a density of 12 500 cells/cm^2^ on a gelatin coated 24 well plate in the mouse MSC media. After 24 h, the media was changed followed by PyVTs being detached from plates, spun down, and resuspended at 10 000 cells/*μ*l in R buffer with the pBs185 CMV-Cre plasmid (Addgene) added at 8 *μ*g/10^6^ cells. PyVTs were electroporated using the following electroporation protocol: 1600 V, 20 ms, and 1 pulse. Cre_PyVTs were plated at a density of 40 000 cells/cm^2^ on top of the MSCs. Co-cultures were maintained in the mMSC media.

### Assessment of the hybrid frequency *in vitro*

Hybrids of co-cultures were identified by locating the GFP positive signal via fluorescence microscopy (Leica DMi8). Cells were only counted if they appeared to be attached, spread, and healthy as electroporation can result in significant cell death. Cells that appeared to be in the late stages of apoptosis were also excluded from the total cell count. Cells that did not exceed the fluorescence intensity of negative control cultures or did not contain at least one nuclei were also excluded from the total count.

### RNASeq

Co-cultures of human cells (hMSCs/T47Ds) were established as described earlier. Cells were detached from the plate and taken to the University of Minnesota's University Flow Cytometry Resource (UFCR) for cell sorting. Gates were set to eliminate dead cells, and GFP positive cell regions were defined based on control co-cultures not containing the floxed GFP reporter. The gene expression analysis was performed with Galaxy software [Minnesota Supercomputing Institute (MSI), University of Minnesota, Minneapolis, MN]. Reads averaged 465 K per samples and were processed and aligned to the human reference genome (hg19) using Tophat (version 2.0.12, open source software, http://ccb.jhu.edu/software/tophat/index.shtml). See supplementary material, Table 1 for information about the total number of reads and percent concordant mapped reads for each cell. The default options supplied with the software were used, and the aligned read files produced by Tophat were processed using Cufflinks software (version 2.2.1, open source software, http://cole-trapnell-lab.github.io/cufflinks/), for further analysis, including assembling transcripts and estimating their abundance. Read counts were normalized to FPKM according to the gene length and total mapped reads. Differential gene expression was determined using the Single Cell Differential Expression (SCDE) toolset. (Differential expression results are provided in supplementary material, Table 1, and SCDE was referenced from Freeman *et al.*^20^) Genes with a P value of less than 0.05 were considered “differentially expressed” and further analyzed for gene ontology (supplementary material, Table 2). Cells were removed from the study if they did not meet the quality check performed by the SCDE clean counts function. For this study, the minimum library size was set to 1000, the minimum reads set to 1 and the minimum detected set to 1. This means that the inclusion criteria included cells in which at least 1000 genes were found, genes which were read at least once and genes which were found in at least one cell. Gene ontology and Kyoto Encyclopedia of Genes and Genomes (KEGG) pathway enrichment analyses were performed with DAVID informatics resources 6.7 of the National Institute of Allergy and Infectious Diseases (NIAID) and the National Institutes of Health (NIH). Gene Cluster Analysis Up-stream filtering of the data was done in the SingulaR package. A threshold of 1 FPKM was set as the limit of detection. Over 26 000 genes with an FPKM >1 in at least one sample were used to obtain the HC analysis. Average linkage hierarchical clustering of the gene expression intensity was performed using the Pearson distance to measure the distance between the gene and single cells. SingulaR (Fluidigm, San Francisco, CA) was used to compute and create the hierarchical clustering and principle component analysis plots. For statistical analysis comparison of DNA content and significant gene changes per chromosome, a normal distribution was assumed, and one-way analyses of variance and post-hoc test (Least Significant Difference, LSD) were used. Data were analyzed with Microsoft Excel (Microsoft, Redmond, WA, USA). RNA-seq data were analyzed with the Cuffdiff or SingulaR programs.

### Assessment of hybrid migration *in vitro*

Co-cultures for time-lapse microscopy were initiated as described in the protocol earlier. Control wells were additionally set up containing either Cre_PyVTs or FloxGFP_mMSCs. After plating, cells were allowed to adhere for 4 h at 37 °C under static conditions before plates were moved to a Citation 3 plate reader (BioTek, Winooski, VT, USA) and imaged under a bright field and a GFP filter every 30 min. Each well was imaged at 10× in an 8 × 8 section while being maintained at 5% CO_2_ and 37 °C. The media was changed after 24 h and then every 48 h thereafter. Images from tracking (h 24–72) were stacked, and fusion products were identified as described in the protocol earlier. The manual tracking tool in NIH Fiji software (ImageJ; National Institutes of Health, Bethesda, MD, USA) was used to determine the paths of migrating cells. These tracks were then analyzed by using the chemotaxis and migration tool in NIH Fiji. The approximate center of the cell was selected in each frame, and the resulting path was used to calculate the velocity, total distance, and directionality. Hybrids of co-cultures were compared to parental cells of the single cell cultures.

### Injection of tumor cells or hybrids into Flox-luc (005125-FVB.129S6(B6)-*Gt(ROSA)26Sor^tm^*^1*(Luc)Kael*^/J) mice

Ethics approval for all animal studies was obtained from the University of Minnesota, Institutional Animal Care and Use Committee (Protocol Nos. 1304-30524A and 1607-33984A). Cells were injected into fat pad #1 after shaving the area and wiping with an alcohol swab, allowing the area to dry prior to injection. Two types of injections were used for this study. The PyVT only injections (Cre_PyVT) included injecting a total of 1000 PyVT cells. The co-culture injections (Co) included injecting 2000 Cre_PvVT cells and mMSCs, with the mMSCs at a ratio of 1:1 with the Cre_PyVT cells. The cells were suspended in 25 *μ*l of the DMEM/F12 (1:1) media and mixed with 25 *μ*l of matrigel (126-2.5, Sigma-Aldrich) and put into a syringe (349606, McKesson, San Francisco, CA) for injection. The syringe was inserted into fat pad #1, and the cells were slowly dispersed into the fat pad. Once cells were dispersed into the fat pad, the syringe was removed and disposed into a sharps container.

### IVIS imaging of mice with tumor and metastases formation

Mice received an intraperitoneal (IP) injection of d-luciferin (122796, Caliper Life Sciences by PerkinElmer, Waltham, MA) and were placed in an induction chamber containing oxygen and isoflurane (NDC 66794-017-25, Piramal, Mumbai, India) (at an induction rate of 1%–5%). After mice were non-responsive to toe pinch, they were placed in the IVIS Spectrum machine (Caliper Life Sciences) while maintaining gas anesthesia (XGI-8, Xenogen by Caliper Life Sciences) with oxygen and 1%–3% isoflurane. Mice were imaged with the IVIS at 5, 10, and 15 min post d-luciferin injection. The exposure time was set to 1 min for all images. Mice were imaged at days 1, 4, 7, 14, 21, 28, and monthly thereafter. The mice were then imaged one final time prior to euthanizing. The IVIS images were analyzed using the Living Image software at the University of Minnesota's University Imaging Center.

### Necropsy and tissue preservation

Primary tumor and lung tissues were removed from mice. Small sections of tissue were taken and flash frozen in liquid nitrogen to be used for RNA extraction. Most of the tissue was placed into a tissue cassette and put into 10% buffered formalin (HT50126-40, Sigma by Sigma-Aldrich) for 24 h to fix for staining. The tissue was then transferred to 30% sucrose (8360-06, Macron Fine Chemicals) for approximately 48 h prior to freezing in optimal cutting temperature (OCT) compound (4583, Tissue-Tek by Sakura, Osaka, Japan) and sectioning with a cryostat (CM1900, Leica, Wetzlar, Germany) at a temperature of approximately −20 °C in sections 10 *μ*m thick. Small pieces were also saved using cork/pin preservation. For cork and pin preservation, a pin was stuck through the cork, parallel to the flat plane of the cork, and a small amount of OCT was placed onto the flat surface. The tissue sample (no greater than 5 mm in diameter) was placed onto the OCT. The tissue on the cork and pin was placed into a labeled cryopreservation tube filled with 1.5 ml of isopentane, sealed, and placed into liquid nitrogen until moved to long-term storage at −80 °C.

### Hematoxylin and Eosin (H&E) Staining

H&E staining was conducted on frozen tissue sections. Tissue sections were allowed to dry upon removal from the freezer before being processed, and hematoxylin (411116, Acros) was filtered before every use. Slides were allowed to sit in hematoxylin between 30 and 90 s. This time was determined before each staining section by using control slides for color quality. Slides were moved to tap water (running) for 2 min and then dipped in the working eosin solution (17372, Acros) for 2 times. The slides were then dehydrated for mounting using the following steps: 15 dips each in 95% ethanol, 100% ethanol (1), 100% ethanol (2), xylene (1) (8668, Macron), and xylene (2). Following dehydration, permount (SP15, Fisher Scientific) was added to the slides and covered with a cover slip. Slides were imaged on a Leica DMi8.

### Immunofluorescence staining

Immunofluorescence staining was conducted on frozen tissue sections. Tissue sections were blocked for 2 h in BGST [5 g bovine serum albumin (BSA) (126575-10GM, Calbiochem by EMD Millipore), 1 g glycine (G7403-100G, Sigma by Sigma-Aldrich), 100 *μ*L Triton X-100 (T8787-100ML, Sigma), 10 ml of 10× phosphate buffered saline (PBS) (BP229-1, Fisher), and 88 ml water] containing 2% mouse serum (M5905-5mL, Sigma-Aldrich) for 2 h prior to the addition of the primary antibody. The anti-luciferase antibody (NB600-307SS, Novus Biologicals, Littleton, CO) was diluted at a ratio of 1:300 in BGST with 2% mouse serum and was kept on the tissue for 12–18 h in the fridge at 4 °C. The tissue sections were washed for 5 min in 1× PBS for 3 times. The secondary antibody, goat anti-mouse IgG Alexa Fluor 647 (A21236, Invitrogen by Thermo-Fisher) was diluted at a ratio of 1:300 in BGST with 2% mouse and 2% goat serum (191256, Sigma-Aldrich) and incubated for 12–18 h. The secondary was added to the tissue sections and left to incubate at room temperature for 2 h. After incubation, the tissue samples were washed for 5 min in 1× PBS for 3 times. DAPI/1,4-Diazabicyclo[2.2.2]octane (DABCO) [1.25 g DABCO (D27802-100G, Sigma-Aldrich), 25 ml glycerol (BP229-1, Fisher by Thermo Fisher), 25 ml of 2× PBS, and 2.5 *μ*l of DAPI (D9542-MG, Sigma-Aldrich)] was added onto the slides before placing a coverslip over the tissue. Coverslips were then sealed and imaged.

### Quantitative real time PCR

mRNAs were extracted from control cells lines (mMSC, PyVTs, MCF7 + luciferase: 2D cultures), control tissues (untreated fat pad and lung), and experimental tissues using the RNA PureLink Kit (Invitrogen). Complementary DNA (cDNA) was synthesized using the High Capacity RNA-to-cDNA Kit (Applied Biosystems, ThemoFisher Scientific). Following the cDNA synthesis, cDNA was treated with DNase using the RNase-Free DNase Set (Qiagen). Primer sequences were GAPDH forward, TGTGTCCGTCGTGGATCTGA; GAPDH reverse, TTGCTGTTGAAGTCGCAGGAG; Firefly luciferase forward, CTCACTGAGACTACATCAGC; Firefly luciferase reverse, TCCAGATCCACAACCTTCGC.^48^ PCR was completed using Power SYBR^®^ green PCR Master Mix (appliedbiosystems, ThemoFisher Scientific) on a LightCycler 96 (Roche) quantitative PCR machine. Ct values were used to determine relative Firefly Luciferase expression as compared to GAPDH for all samples.

## SUPPLEMENTARY MATERIAL

See supplementary material for flow sorting parameters used to isolate human hybrids for RNAseq (supplementary Fig. 1), phenotypic characterization of mMSCs prior to execution of *in vitro* and *in vivo* studies (supplementary Fig. 2), immunofluorescence and FISH staining of hybrids (supplementary Fig. 3), migration of hybrids relative to parental cells of co-cultures (supplementary Fig. 4), and immunofluorescence staining of luciferase expressing control cells and control tissues (supplementary Fig. 5).
